# Context *v.* algorithm: evidence that a transdiagnostic framework of contextual clinical characterization is of more clinical value than categorical diagnosis

**DOI:** 10.1017/S0033291721003445

**Published:** 2023-04

**Authors:** Jim van Os, Lotta-Katrin Pries, Margreet ten Have, Ron de Graaf, Saskia van Dorsselaer, Maarten Bak, Gunter Kenis, Bochao D. Lin, Nicole Gunther, Jurjen J. Luykx, Bart P. F. Rutten, Sinan Guloksuz

**Affiliations:** 1 Department of Psychiatry and Neuropsychology, School for Mental Health and Neuroscience, Maastricht University Medical Centre, Maastricht, The Netherlands; 2 Department of Psychiatry, UMC Utrecht Brain Center, University Medical Center Utrecht, Utrecht University, Utrecht, The Netherlands; 3 Department of Psychosis Studies, Institute of Psychiatry, Psychology & Neuroscience, King's College London, London, UK; 4 Department of Epidemiology, Netherlands Institute of Mental Health and Addiction, Utrecht, The Netherlands; 5 FACT, Mondriaan Mental Health, Maastricht, The Netherlands; 6 Department of Translational Neuroscience, UMC Utrecht Brain Center, University Medical Center Utrecht, Utrecht University, Utrecht, The Netherlands; 7 School of Psychology, Open University, Heerlen, The Netherlands; 8 Department of Neurology, Brain Centre Rudolf Magnus, University Medical Centre Utrecht, Utrecht, The Netherlands; 9 GGNet Mental Health, Apeldoorn, The Netherlands; 10 Department of Psychiatry, Yale University School of Medicine, New Haven, CT, USA

**Keywords:** Diagnosis, mental health, psychosis, depression, symptoms, genetics

## Abstract

**Background:**

A transdiagnostic and contextual framework of ‘clinical characterization’, combining clinical, psychopathological, sociodemographic, etiological, and other personal contextual data, may add clinical value over and above categorical algorithm-based diagnosis.

**Methods:**

Prediction of need for care and health care outcomes was examined prospectively as a function of the contextual clinical characterization diagnostic framework in a prospective general population cohort (*n* = 6646 at baseline), interviewed four times between 2007 and 2018 (NEMESIS-2). Measures of need, service use, and use of medication were predicted as a function of any of 13 DSM-IV diagnoses, both separately and in combination with clinical characterization across multiple domains: social circumstances/demographics, symptom dimensions, physical health, clinical/etiological factors, staging, and polygenic risk scores (PRS). Effect sizes were expressed as population attributable fractions.

**Results:**

Any prediction of DSM-diagnosis in relation to need and outcome in separate models was entirely reducible to components of contextual clinical characterization in joint models, particularly the component of transdiagnostic symptom dimensions (a simple score of the number of anxiety, depression, mania, and psychosis symptoms) and staging (subthreshold, incidence, persistence), and to a lesser degree clinical factors (early adversity, family history, suicidality, slowness at interview, neuroticism, and extraversion), and sociodemographic factors. Clinical characterization components in combination predicted more than any component in isolation. PRS did not meaningfully contribute to any clinical characterization model.

**Conclusion:**

A transdiagnostic framework of contextual clinical characterization is of more value to patients than a categorical system of algorithmic ordering of psychopathology.

## Introduction

Diagnosis in psychiatry represents an unresolved issue (Guloksuz & van Os, 2020). Although the practice of classification according to ICD and DSM criteria remains firmly rooted in clinical practice, it is widely recognized that the classical diagnostic functions of predicting the need for care and health care outcome are not well served in the ICD/DSM diagnostic framework (Mullins-Sweatt, Lengel, & DeShong, 2016). Research shows that individuals in the same ICD/DSM diagnostic category are different with regard to the need for care and health care outcome, and as a group show only weak differences in risk factors, psychopathology, need for care, and outcome compared to patients in other diagnostic categories, as evidenced by low diagnostic likelihood ratios (Allardyce, McCreadie, Morrison, & van Os, 2007; Van Os, 2010; Van Os et al., 1999, 2000).

In fact, it is increasingly recognized that mental suffering may be *transdiagnostic* and *contextual*. ‘Transdiagnostic’ refers to the growing awareness that symptoms are not specific to mental disorders, occurring in highly personal clusters that differ from person to person. ‘Contextual’ refers to the growing awareness that psychopathology arises across a range of contexts including social, existential, somatic, and temporal factors, that also require ‘diagnosing’ to guide clinical approaches (van Os, Delespaul, Wigman, Myin-Germeys, & Wichers, 2013). Thus taking into account transdiagnostic and contextual perspectives in diagnosis opens up a new way to advance clinical practice beyond the traditional psychiatric taxa (Dalgleish, Black, Johnston, & Bevan, 2020). Although a range of alternative, more ‘transdiagnostic’, diagnostic formulations have been proposed, for example, dimensional alternatives such as Hierarchical Taxonomy of Psychopathology (HiTOP) (Kotov et al., 2017) and Research Domain Criteria (RDoC) (Cuthbert & Insel, 2010), as well as network models of psychopathology (Borsboom & Cramer, 2013), these were developed and researched mostly from the perspective of contribution to academic theory, not of contribution to patient value, i.e. adding to the diagnostic prediction of care need and health care outcome. In addition, they do not offer a contextual perspective. Thus, diagnostic innovation with a specific focus on adding patient value in the ‘moral’ era of medicine and health care (Berwick, 2016) may require a different approach (van Os, Guloksuz, Vijn, Hafkenscheid, & Delespaul, 2019).

A more patient-centered and contextual approach toward diagnostic innovation is the ‘clinical characterization’ diagnostic framework (Maj, 2020). Clinical characterization is focused on specifically increasing patient value by combining or replacing categorical ICD/DSM diagnosis with transdiagnostic, personalized, and contextual information on symptoms, clinical severity, clinical staging (McGorry & van Os, 2013), and antecedent and concomitant variables (Maj, 2020). While clinical characterization will always form part of the assessment process, the specific inclusion of clinical characterization in the diagnostic framework opens up a way of specifically researching and validating modes of clinical characterization, and how these may maximize patient value and reduce the self-perpetuated oversized importance attached to context-less categorical and algorithm-based classification *per se* (van Os et al., 2019).

Here, we wished to examine the value of the diagnostic framework of clinical characterization by specifically examining the transdiagnostic contextual components of the framework in terms of contribution to predicting care need and health care outcome, in comparison with the categorical algorithm-based approach. To this end, we prospectively modeled the occurrence of care needs and outcomes associated with transdiagnostic psychopathology in a unique representative general population cohort interviewed four times over a period of 9 years. As there is much speculation about including biological information in the clinical characterization component, particularly genetic information in the form of polygenic risk scores (PRS; Murray et al., 2021), these were also included in the analyses.

## Method

### Sample

All four waves of the Netherlands Mental Health Survey and Incidence Study-2 (NEMESIS-2) were used. NEMESIS-2 was conducted to study the prevalence, incidence, course, and consequences of mental disorders in the Dutch general population (*n* = 6646 at baseline). The baseline data of NEMESIS-2 were collected from 2007 to 2009, follow-up was until 2018. Non-clinician, trained interviewers applied the Composite International Diagnostic Interview (CIDI) version 3.0 (Alonso et al., 2004; de Graaf, ten Have, Burger, & Buist-Bouwman, 2008) and additional questionnaires during home visits. Further details are provided in the online Supplementary material.

### Assessment of DSM-IV disorders

The following 13 CIDI, version 3.0, DSM-IV diagnoses were assessed: major depression, dysthymia, bipolar disorder, panic disorder, agoraphobia, social phobia, specific phobia, GAD, alcohol abuse and dependence, drug abuse and dependence, and any clinical psychosis. Further details of the diagnostic procedure are provided in the online Supplemental material.

### Outcomes to be predicted by diagnostic framework

#### Mental health service use

Service use was measured based on the service use section of NEMESIS (Bijl & Ravelli, 2000) at each interview wave. A variable of receiving any mental health care was defined on the basis of any care received for mental health or addiction problems by psychiatrists, psychologists, psychotherapists, and professionals working in mental health and addiction care, in the last 12 months (T0) or since the last interview (T1–T3).

#### Unmet need for care and medication use

Unmet need for care was defined as the person reporting, at each interview wave, that he or she needed care for a mental problem but had not sought such care, or not enough, in the last 12 months. At each wave, a binary rating was made for use of any medication for mental problems or addiction in the last 12 months.

### Clinical characterization components

#### Staging variables

Staging variables were constructed to create a variable providing a temporal sub-characterization to the psychopathological variables in the model. Four mutually exclusive staging variables were constructed, at each wave, indicating *no disorder*, *subthreshold syndrome*, *incident disorder*, and *persistent disorder*. Variables were mutually exclusive in that persistent disorder trumped incident and subthreshold, and incident disorder trumped subthreshold syndrome. Significant subthreshold psychopathology was considered present if individuals had rated positive on any CIDI 3.0 core screening symptom (for each disorder, CIDI 3.0 has a two-stage process of core symptom screening questions which, if positive, result in follow-up symptom questions) of any anxiety disorder, depression, or bipolar disorder, or had any self-reported psychotic experience in the absence of any diagnosis of, respectively, any anxiety disorder, depression, bipolar disorder, or clinical psychosis. Incident disorder was defined as the first occurrence of any of the 13 disorders, defined above, over the NEMESIS-2 period of observation. Baseline occurrence was considered as ‘incident’ if the first onset had transpired in the 6 months before baseline interview, based on the age of onset questions about the disorder. Persistence was defined as any occurrence of a DSM-IV disorder persisting from one interview wave to the next.

#### Sociodemographic variables

Demographic variables included were *sex* (0 = male, 1 = female), age in years, and dichotomous *ethnic minority status* (Moroccan, Turkish, Surinamese, Antillean, Indonesian, or other non-Western ethnic groups). *Age* was analyzed as a dichotomous variable, defining a younger age group encompassing the range most at risk of the onset of a psychotic disorder (18–35 years, 24% at baseline) *v.* the older group, consistent with previous work in this sample (Hasmi et al., 2021). *Marital status* at each interview was coded married/widowed *v.* divorced/never married. *Unemployment* at each interview was coded as being unemployed or disabled *v.* employed/student/homemaker/retired. *Educational level* at baseline was a two-level variable (primary, lower, and higher secondary *v.* higher professional/university education). *Income* at each interview was net annual household income (i.e. individual and, if applicable, partner), rated on a scale from 1 to 14 (not rated at one interview and predicted linearly from the values at the interviews before and after) and modeled as ‘low income’ *v.* other, by dichotomizing around the 25% percentile. Having ever been on *disability benefit* over the period of observation was analyzed as a binary variable. The variable ‘*living alone*’ at each interview indicated that the participant was the only person in the household. The variable ‘*children at home*’ indicated the presence of one or more children in the household. The *perceived status gap* was assessed at T1, T2, and T3 using two questions. First, the MacArthur Scale of Subjective Social Status (Adler, Epel, Castellazzo, & Ickovics, 2000) was used to rate subjective social status. In an easy pictorial format, it presents a ‘social ladder’ with 10 levels and asks individuals to place an ‘X’ on the rung on which they feel they stand. The second question was about a similar ladder, but this time with regard to the desired level of social status. The difference between the subjective desired and actual social status was used as an independent variable in the analyses. The mean value of T1–T3 was used to replace missing values at T0. In the analyses, it was dichotomized around the perception of being more than one level below desired social status. *Level of urbanicity of current residence*, assessed at each interview wave, was defined at five levels based on the Dutch classification of residence topography or population density: (1) countryside (distances to facilities is larger), (2) village (<25 000 inhabitants), (3) small city (25 000–50 000 inhabitants), (4) medium city (50 000–100 000 inhabitants), (5) large city (>100 000 inhabitants). Consistent with previous work, the cut-off of at least >50 000 inhabitants was used to define the binary variable of urban area (Guloksuz et al., 2015).

#### Clinical variables

*Childhood adversity* was assessed at T0 using a questionnaire based on the NEMESIS trauma questionnaire (de Graaf, Ten Have, & van Dorsselaer, 2010). Whenever a subject reported having experienced one of five types of childhood adversity before the age of 16 years [emotional neglect, physical abuse, psychological abuse, peer victimization (bullying), and sexual abuse], they were asked to state how often it had occurred. From this, as described previously, a childhood adversity sum score was derived and dichotomized at the 80th percentile (Heins et al., 2011; van Dam et al., 2015; van Os, Marsman, van Dam, Simons, & Investigators, 2017). Based on the ‘Brugha Life events section’ (Brugha, Bebbington, Tennant, & Hurry, 1985), participants were asked at each interview whether they experienced one of 10 negative *life events* within the last 12 months at T0 and T3, and some additional pregnancy- and illness-related negative life events at T1–T2. Examples of items are serious sickness, death of a family member or close friend, and serious financial problems. A dichotomous exposure was created around at least one negative life event in the last year. *Family history* was assessed as a person-level binary variable in two stages, as described previously (Radhakrishnan et al., 2019) and detailed in the online Supplementary material. One of the interviewer observations that was rated at each interview concerned the degree to which the person had understood the interview questions or was ‘*slow to understand*’ (very good, reasonable, poor). Any rating of understanding the questions less than very good at any of the interviews was rated as a dichotomous variable of ‘slow to understand’. For each interview, a binary variable ‘*any suicidality*’ was rated positive if the participant had admitted to any suicidal plans, thoughts, or attempts at the corresponding items of the CIDI interview. Two 12 binary item subscales of the Eysenck Personality Questionnaire – Revised Short Scale (EPQ-RSS) were used at baseline to measure *neuroticism* (i.e. emotional instability, worrying, nervousness) and *extraversion* (i.e. impulsivity, outgoing, lively) (Schotanus-Dijkstra et al., 2016). For each personality trait, a total score was computed with higher scores indicating higher levels of the trait ranging from 0 to 12 (Cronbach's *α* 0.80 for neuroticism and 0.83 for extraversion), analyzed as binary variables, dichotomized around the 75th percentile.

#### Symptoms scores

Scores of depressive (28 symptoms), anxiety (43 symptoms), mania (18 symptoms), and psychosis symptoms (20 symptoms) were based on adding the relevant CIDI 3.0 symptoms as described previously in detail (van Nierop et al., 2015).

#### Physical health variables

*Hearing impairment* and *visual impairment* were assessed during the face-to-face interview at each wave, by asking whether participants had experienced deafness or serious hearing impairment, or serious visual impairment in the past 12 months. Ratings were yes (1) or no (0). *High BMI*, assessed at each interview, was defined as BMI > 25. *Physical exercise*, assessed at each interview, was coded ‘1’ if the person met the Dutch health criterion for sufficient physical activity and otherwise coded ‘0’. *Any smoking* of tobacco, assessed at each interview, was coded ‘1’ and otherwise coded ‘0’. Binary *presence of pain* was assessed at each interview with The Short-Form-36 Health Survey (SF-36) (Stewart & Ware, 1992) pain module, dichotomized around the 75% percentile. The presence of 15 groups of *somatic disorder* was assessed at each interview and coded as ‘1’ for any presence of one or more somatic disorders – and otherwise coded ‘0’.

#### Polygenic risk scores for schizophrenia, bipolar disorder, depression, and IQ

The *polygenic risk score for schizophrenia* (PRS-SZ) was created from best-estimate genotypes at six different *p* thresholds (0.5, 0.1, 0.05, 5.10^−3^, 5.10^−5^, 5.10^−8^). For our primary analyses, we used the *p* threshold of <0.05, as this threshold explained most variation in liability in the Psychiatric Genomics Consortium analysis (Schizophrenia Working Group of the Psychiatric Genomics Consortium, 2014) and was shown to perform well for the current phenotype of SF-36 mental health (Pries et al., 2020). Further details on the genotyping procedure and PRS calculation were described previously indetail (Guloksuz et al., 2020; Pries et al., 2018). Consistent with previous analyses, statistical analyses with PRS-SZ were adjusted for three principal components (Pries et al., 2020).

Material for DNA analysis of sufficient quality, and hence for PRS calculation, was available for 3104 individuals (47%) at T0. Excluding individuals who at interview has been assessed as a member of an ethnic minority, given lack of generalizability of PRS to this group, and individuals diagnosed with a clinical psychosis, left 3052 for PRS calculation.

#### DSM-IV diagnosis

CIDI 3.0 lifetime diagnoses (T0) and interval diagnoses (T1-T3) of the 13 disorders described above served as the diagnostic variables. Rates presented are not weighted and therefore may slightly differ from previous results (de Graaf, ten Have, van Gool, & van Dorsselaer, 2012).

### Analysis

#### Logistic regression models of three dependent variables and 46 independent variables representing six domains of clinical characterization

All analyses were performed using Stata, version 16 (StataCorp, 2019). *p* < 0.05 (two-tailed) was considered nominally statistically significant. We tested the contributions of 46 independent variables, clustered into six clinical characterization domains, entering the variables pertaining to each specific group (DSM diagnosis, symptoms, social, clinical, physical, and staging), in logistic regression models of incidence of three binary dependent variables relevant for diagnostic function: unmet need for care, use of mental health service, and use of medication.

**Fig. 1.**
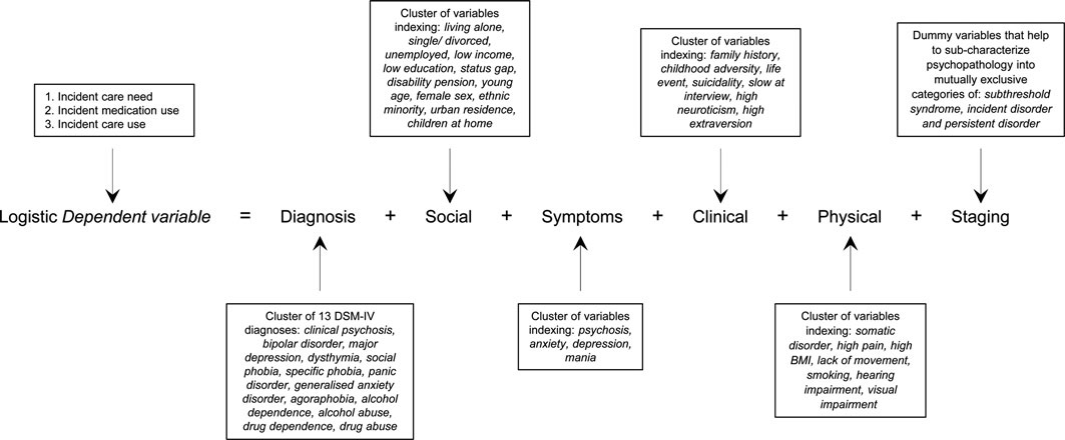
Main logistic regression model of three dependent variables and 46 independent variables, representing six domains of clinical characterization.

We first calculated the contribution of the independent variables pertaining to the six domains of clinical characterization separately, in separate logistic regression models for each of the three dependent variables. This was followed by testing the contributions of the independent variables pertaining to the six domains entered jointly into a single model, for each of the three dependent variables. This latter full logistic regression model is visualized in Fig. 1.

In order to understand the interplay between specifically the domain of DSM diagnosis in relation to each of the other five domains, we separately modeled the joint contribution of the diagnosis domain and each of the other five domains.

#### Definition of ‘incidence’ of the three dependent variables

The NEMESIS-2 repeated cross-sectional design (i.e. each person contributing four observations over time) allowed fitting multiple prediction models for binary outcomes (i.e. care need, medication use, care use) using logistic regression to model the incidence of the outcome at time point *t*. The outcome was considered incident if there was the absence of the outcome at the previous wave *t–1*, and the presence of the outcome at time point *t*.

The risk set for the three outcomes can be seen in Table 1 (left side; incidence).

**Table 1.** Distribution of need for care and health care outcomes, per interview wave, in NEMESIS-2 cohort, incident, and prevalent

Wave		Care need	Medication use	Service use	Care need	Medication use	Service use
		Incidence^a^			Prevalence		
1	%	0	0	0	2.00%	6.40%	6.60%
	*N*	6506	6506	6506	6506	6506	6506
2	%	2.50%	3.00%	7.50%	2.80%	6.20%	11.00%
	*N*	5303	5303	5303	5303	5303	5303
3	%	1.80%	3.20%	5.50%	2.20%	6.30%	9.90%
	*N*	4618	4618	4618	4618	4618	4618
4	%	2.20%	2.70%	6.30%	2.70%	5.90%	10.00%
	*N*	4007	4007	4007	4007	4007	4007
Total	%	1.50%	2.00%	4.40%	2.40%	6.20%	9.30%
	*N*	20 434	20 434	20 434	20 434	20 434	20 434

a Incidence was defined as the absence of the outcome at the previous wave *t–1*, and the presence of the outcome at time point *t*, over the four interview waves in NEMESIS-2.

The Stata *cluster* option was used to take into account intra-group correlations occasioned by clustering of observations within individuals.

#### Testing the contribution of variables clustered in six domains of clinical characterization

Contributions to the model were evaluated, for each of the clusters of variables representing a domain of clinical characterization, with the population attributable fraction (PAF) for binary outcomes. The Stata PUNAF command was used (Newson, 2013) to estimate the attributable fraction and the PAF with 95% CIs for each cluster of variables representing a domain of clinical characterization. Under the assumption that the different risk groups are causally associated with the outcome, the PAF indicates the proportion of the care need (or other outcome used) that might be prevented if the risk were eliminated (Levine, 2007).

#### PRS subsample

Given the fact that polygenic risk was only present in a subsample, separate analyses were conducted with the five clinical characterization variable groups with polygenic risk variables added as a seventh group (polygenic risk, diagnosis, symptom, social, clinical, physical, and staging) on the same three outcomes, in order to evaluate the contribution of PRS. Models including PRS were adjusted for three principal components, conform previous work (Guloksuz et al., 2020; Marsman et al., 2020; Pries et al., 2020).

#### Overfitting and multicollinearity

Given the use of up to 46 independent variables in the logistic regression model, we examined the possibility of overfitting and multicollinearity that may ensue. This is described further in the online Supplementary material.

## Results

Distributions of the clinical characterization variables over the four waves are shown in online Supplementary Tables S1–S5. The distribution of the outcomes, incident and prevalent, is shown in Table 1. In the subsample of 3052 with PRS data, the mean value of PRSZ-SZ was −131.2 (s.d. = 4.3).

In the models of clinical characterization domains *separately* predicting need, medication use, and mental health service use, diagnosis and clinical characterization groups all contributed, with somewhat weaker contribution of the social and physical clinical characterization variables. In the models of clinical characterization domains *jointly* predicting need, medication use, and mental health service use, particularly symptom and staging variables contributed to all predictions whereas diagnosis variables did not predict any outcome and other variables had more specific contributions (e.g. social variables to need and service use, and clinical variables to need and medication use). The physical domain did not contribute meaningfully (Table 2). Also, for all models, the combined contribution of clinical characterization variables in the models examining the contributions of the domains jointly was around 2–2.5 times larger than the contribution of categorical diagnosis in the models examining the contributions of the domains separately (Table 2).

**Table 2.** Contribution of diagnosis and clinical characterization variable groups, modeled separately and jointly, to models of need incidence (need for care, medication, service use)

Care need	*N* observations	*N* persons	PAF^a^ separately^b^	CI^c^ low	CI^c^ high	PAF^a^ jointly^d^	CI^c^ low	CI^c^ high
Full model	13 078	5160				0.66	0.58	0.71
Diagnostic^e^			0.24	0.18	0.28	−0.15	−0.44	0.08
Social^e^			0.30	0.18	0.40	0.15	0.01	0.27
Symptoms^e^			0.28	0.23	0.33	0.25	0.16	0.34
Clinical^e^			0.40	0.32	0.47	0.30	0.20	0.38
Physical^e^			0.11	0.06	0.15	0.04	−0.01	0.09
Staging^e^			0.61	0.53	0.68	0.51	0.39	0.60
Medication use								
Full model	12 558	5034				0.60	0.53	0.65
Diagnostic^e^			0.32	0.27	0.36	−0.01	−0.19	0.15
Social^e^			0.14	0.04	0.23	0.02	−0.08	0.12
Symptoms^e^			0.38	0.32	0.42	0.38	0.30	0.44
Clinical^e^			0.32	0.25	0.38	0.17	0.09	0.25
Physical^e^			0.11	0.07	0.15	0.04	0.01	0.08
Staging^e^			0.57	0.50	0.63	0.38	0.25	0.49
Mental health care use								
Full model	12 200	5040				0.59	0.54	0.63
Diagnostic^e^			0.27	0.24	0.30	0.05	−0.03	0.13
Social^e^			0.21	0.15	0.28	0.12	0.05	0.18
Symptoms^e^			0.32	0.28	0.35	0.28	0.22	0.33
Clinical^e^			0.21	0.17	0.25	0.05	0.01	0.10
Physical^e^			0.04	0.03	0.06	0.01	−0.01	0.02
Staging^e^			0.59	0.54	0.63	0.41	0.33	0.48

a Population attributable fraction: proportion of the outcome that could have been prevented had the area of clinical characterization been reduced to zero.

b PAF separately – the variable group of each domain was modeled separately, yielding PAF for each domain separately.

c CI, confidence interval.

d PAF jointly – the variable groups of all domains were modeled jointly, yielding mutually adjusted PAF.

e The following six clinical characterization variable clusters were examined for contribution to the model:

*Diagnostic:* cluster of 13 DSM-IV diagnoses (clinical psychosis, bipolar disorder, major depression, dysthymia, social phobia, specific phobia, panic disorder, generalized anxiety disorder, agoraphobia, alcohol dependence, alcohol abuse, drug dependence, drug abuse).

*Social:* cluster of variables indexing: living alone, single/divorced, unemployed, low income, low education, status gap, disability pension, young age, female sex, ethnic minority, urban residence, children at home.

*Symptoms:* cluster of variables indexing: psychosis, anxiety, depression, mania.

*Clinical:* cluster of variables indexing: family history, childhood adversity, life event, suicidality, slow at interview, high neuroticism, high extraversion.

*Physical:* cluster of variables indexing: somatic disorder, high pain, high BMI, lack of movement, smoking, hearing impairment, visual impairment.

*Staging:* three dummy variables that help to sub-characterize psychopathology into mutually exclusive categories of: subthreshold syndrome, incident disorder, and persistent disorder.

In the models examining the contribution of the diagnosis domain in combination with each of the other four domains separately (Table 3), it is revealed that any contribution of the diagnosis domain was reducible to the symptom domain. Similarly, the diagnosis domain did not contribute much over and above the contribution of the staging domain. The contribution of the diagnosis domain was mostly complementary to the social and the clinical domain, and trumped the contribution of the physical domain (Table 3).

**Table 3.** Clinical characterization domain of DSM diagnosis modeled jointly with each of the other five domains, for three outcomes

Clinical characterization domain^a^	Care need			Medication use			Mental health care use		
	PAF^b^	CI^c^ low	CI^c^ high	PAF	CI^c^ low	CI^c^ high	PAF	CI^c^ low	CI^c^ high
Full model^d^	0.31	0.26	0.36	0.40	0.35	0.44	0.34	0.31	0.38
Diagnostic^e^	0.08	−0.08	0.21	0.09	−0.03	0.19	0.08	0.02	0.14
Symptoms^e^	0.29	0.24	0.34	0.37	0.33	0.42	0.31	0.27	0.35
Full model^d^	0.61	0.53	0.68	0.55	0.48	0.61	0.56	0.52	0.61
Diagnostic^e^	0.15	0.04	0.25	0.28	0.21	0.34	0.26	0.22	0.29
Staging^e^	0.59	0.51	0.66	0.49	0.39	0.56	0.47	0.40	0.53
Full model^d^	0.44	0.37	0.51	0.43	0.36	0.48	0.34	0.30	0.37
Diagnostic^e^	0.23	0.18	0.28	0.30	0.25	0.35	0.27	0.24	0.30
Clinical^e^	0.38	0.29	0.45	0.27	0.20	0.33	0.13	0.09	0.17
Full model^d^	0.40	0.29	0.49	0.36	0.27	0.44	0.41	0.34	0.46
Diagnostic^e^	0.25	0.19	0.30	0.31	0.27	0.36	0.27	0.25	0.30
Social^e^	0.25	0.12	0.35	0.09	−0.01	0.18	0.16	0.10	0.22
Full model^d^	0.28	0.23	0.33	0.35	0.30	0.40	0.29	0.26	0.31
Diagnostic^e^	0.24	0.19	0.29	0.31	0.27	0.36	0.27	0.24	0.30
Physical^e^	0.08	0.03	0.12	0.08	0.05	0.12	0.02	0.01	0.03

a The following six clinical characterization variable clusters were examined for contribution to the model:

*Diagnostic:* cluster of 13 DSM-IV diagnoses (clinical psychosis, bipolar disorder, major depression, dysthymia, social phobia, specific phobia, panic disorder, generalized anxiety disorder, agoraphobia, alcohol dependence, alcohol abuse, drug dependence, drug abuse).

*Social:* cluster of variables indexing: living alone, single/divorced, unemployed, low income, low education, status gap, disability pension, young age, female sex, ethnic minority, urban residence, children at home.

*Symptoms:* cluster of variables indexing: psychosis, anxiety, depression, mania.

*Clinical:* cluster of variables indexing: family history, childhood adversity, life event, suicidality, slow at interview, high neuroticism, high extraversion.

*Physical:* cluster of variables indexing: somatic disorder, high pain, high BMI, lack of movement, smoking, hearing impairment, visual impairment.

*Staging:* Dummy variables that help to sub-characterize psychopathology into mutually exclusive categories of: subthreshold syndrome, incident disorder, and persistent disorder.

b Population attributable fraction: proportion of the outcome that could have been prevented had the area of clinical characterization been reduced to zero.

c CI, confidence interval.

d Full model: combined contribution to the model of domain of DSM diagnosis with the other domain in the model.

e Contribution to the model of one domain of clinical characterization, adjusted for the other domain in the model.

### PRS-SZ added to clinical characterization

Analyses with the PRS variable added to the clinical characterization framework showed similar results for the non-PRS clinical characterization variables and only one significant but marginal contribution (PAF = 4%) across the models with the different components included separately and no meaningful or significant contribution in any of the models with the different components jointly included (maximum PAF = 2%; data not shown).

## Discussion

### Findings

The results indicate that although formal categorical diagnoses predict care need and health care outcomes (service use and medication use), this is explained entirely by contextual clinical characterization at the level of the person. In other words, categorical diagnosis in the presence of adequate contextual characterization conveys no additional information whereas the reverse does not hold. It could be argued, therefore, that the diagnostic process should focus on contextual clinical characterization first, rendering the process of formal diagnosis of secondary, administrative importance from the perspective of clinical utility, as the elements in the clinical characterization framework can be translated directly to clinical interventions in a way that formal diagnosis cannot ‘compete’ with (Spitzer, 1998). For example, exposure to childhood adversity, excessive cannabis use, or loss of a relative can directly inform on a specific treatment need in a way that the diagnosis of ‘schizophrenia’ or ‘depression’ cannot.

The results are in agreement with a growing body of evidence that the etiology, treatment, and expression of mental illness is largely transdiagnostic, i.e. better described as overlapping and dynamically evolving personal symptom constellations that transcend classic diagnostic boundaries, arising from overlapping liabilities with little diagnostic specificity (Brainstorm Consortium et al., 2018) and managed with largely non-specific treatment approaches (Dalgleish et al., 2020). In such a transdiagnostic era of psychopathology, clinical characterization is the most fitting and efficient way to predict treatment need and service use outcomes, thus providing value for patients and clinicians.

While it may be argued that good clinical care will always focus on accurate clinical characterization, actual clinical practice in psychiatry continues to attach outsized importance to the ‘correct’ diagnosis, often subjecting each patient to lengthy structured interviews that yield a diagnosis through a series of computerized algorithms – yet are not equipped to yield even simple dimensional scores of major symptom domains. In fact, the influence of formal categorical diagnosis in psychiatry is such that it has been described as an ‘epistemic prison’ (Hyman, 2010). In contrast, a structured format for accurate contextual clinical characterization at the level of the person in routine clinical practice has yet to see the light. Academic studies on how to approach the issue of clinical characterization for major syndromes have only recently begun to appear (Maj et al., 2020).

### Components of contextual clinical characterization

The results suggest that simple contextual clinical measures account for a large proportion of the outcome whereas omnibus measures of common variants of genetic liability do not significantly contribute at all. Although there is much speculation about the clinical use of PRS (Murray et al., 2021), these results are in line with a previous study in the same sample, showing that transdiagnostic PRS did not contribute meaningfully to the onset of psychopathology (Marsman et al., 2020). The finding that PRS did not contribute to the clinical characterization framework is disappointing, albeit not unexpected, given that genetic liability to mental illness is broadly distributed, each individual carrying thousands of small-effect genetic risk variants. Although there have been reports of PRS being associated with clinical outcomes, these associations generally are characterized by statistical significance but tiny effect sizes (Murray et al., 2021). The staging component of clinical characterization had the largest impact on need and outcome and much of the effect of the diagnostic component was reducible to staging. This is not surprising, as persistence of psychopathology over a 3-year follow-up period provides a much greater window of opportunity to seek help and develop need than incident disorder or subsyndromal manifestations. Nevertheless, as a simple measure providing a temporal perspective to psychopathology, it contributes considerably to the utility of clinical characterization. Symptoms clearly ‘trumped’ categorical diagnosis in predicting need and outcome, in line with a large body of research showing quantitative measures of psychopathology outperform categories in representing psychopathology (Haslam, McGrath, Viechtbauer, & Kuppens, 2020). Symptoms also contributed most persistently across the three outcomes, whereas other factors, such as clinical factors and social factors, had more modest and/or less consistent contributions. This suggests that the components of the clinical characterization model may contribute differentially. However, this requires replication across different samples. Physical health variables had the least impact on the models. However, as the range of outcomes under study was limited, these cannot be considered without value. Indeed, somatic health is an important outcome in the mentally ill, and physical variables likely are diagnostically relevant in this domain.

### Psychopathology components of clinical characterization

We included three psychopathology-based domains of clinical characterization that were all derived, albeit in a different fashion, from the same CIDI3.0 items: diagnosis, symptoms, and staging. The findings were consistent in showing poor performance of psychopathology represented as ex-cathedra algorithmic ordering, i.e. DSM diagnoses. Compared to the complex rules of algorithmic decision making, a simple dimensional representation of four-symptom dimensions performed much better, as did the measure of psychopathology in relation to temporality. These findings suggest that the elaborate and time-consuming iterating cycle of development and revision of algorithm-based systems for the diagnosis of psychopathology may not be necessary. Instead, it may be more useful to focus on a system describing contextual components of clinical characterization with direct use for clinical practice.

### Methodological issues

The results should be interpreted in light of several methodological issues. Although the study was prospective, outcomes used were crude as was the measure of staging. On the other hand, this would mimic the actual situation of routine clinical practice in many countries. The analytical paradigm was to analyze the incidence of care need and service use rather than the incidence of psychopathology, instead including psychopathology as a ‘staged’ measure including subthreshold state, incidence, and persistence. This analytical framework was chosen to reproduce the pathway to care as it evolves in natural settings, with the degree of psychopathology, together with a range of moderators as captured by the clinical characterization framework, determine help-seeking behavior, and, eventually, service use (Goldberg & Huxley, 1980). The study was conducted in a small high-income country which will impact outcomes such as service use and use of medication. Therefore, the contribution of clinical characterization variables to these outcomes requires further study in other countries with different health care coverages, particularly low-income and intermediate-income countries.

## Supplementary material

For supplementary material accompanying this paper visit https://doi.org/10.1017/S0033291721003445. click here to view supplementary material
