# Supplementary material for: Context v. algorithm: evidence that a transdiagnostic framework of contextual clinical characterization is of more clinical value than categorical diagnosis
Source: Psychol Med. 2021 Aug 25;53(5):1825–33. doi: 10.1017/S0033291721003445 (PMC10106290; doi:10.1017/S0033291721003445)
Supplement: Supplementary file 1 [file S0033291721003445sup001.pdf]

Supplemental material

Context versus algorithm: Evidence that a transdiagnostic framework of contextual clinical characterization is of more clinical value than categorical diagnosis

Jim van Os, Lotta-Katrin Pries, Margreet ten Have, Ron de Graaf, Saskia van Dorsselaer, Maarten Bak, Gunter Kenis, Bochao D. Lin, Nicole Gunther, Jurjen J. Luykx, Bart P.F. Rutten, Sinan Guloksuz

Table of contents

METHOD **3**

Sample *3*

Assessment of DSM-IV disorders *3*

Overfitting and multicollinearity *4*

Family history *4*

Table S1 – Distribution of 13 DSM-IV diagnoses, per interview wave, in NEMESIS-2 cohort **5**

Table S2. Distribution of factors in social dimension of clinical characterization, per interview wave, in NEMESIS-2 cohort **6**

Table S3. Distribution of factors in clinical dimension of clinical characterization, per interview wave, in NEMESIS-2 cohort **7**

Table S4. Distribution of factors in the somatic dimension of clinical characterization, per interview wave, in NEMESIS-2 cohort **8**

Table S5. Distribution of symptom scores dimension of clinical characterization, per interview wave, in NEMESIS-2 cohort **9**

METHOD

Sample

All four waves of the Netherlands Mental Health Survey and Incidence Study-2 (NEMESIS-2) were used. NEMESIS-2 was conducted to study the prevalence, incidence, course, and consequences of mental disorders in the Dutch general population (n=6646 at baseline). The baseline data of NEMESIS-2 were collected from 2007 to 2009, follow-up was until 2018. Non-clinician, trained interviewers applied the Composite International Diagnostic Interview (CIDI) version 3.0 (Alonso et al., 2004; de Graaf, ten Have, Burger, & Buist-Bouwman, 2008) and additional questionnaires during home visits. Full details of NEMESIS-2 are provided elsewhere (de Graaf, Ten Have, & van Dorsselaer, 2010; de Graaf, ten Have, van Gool, & van Dorsselaer, 2012). To ensure representativeness of the sample in terms of age (between the ages of 18 and 65 at baseline), region, and population density, a multistage random sampling procedure was applied. Dutch illiteracy was an exclusion criterion. The first wave (T0) enrolled 6,646 participants (response rate 65.1%; average interview duration: 95 minutes), who were followed up in 3 visits within 9 years: successive response rates at year 3 (T1), year 6 (T2), and year 9 (T3) were 80.4% (*n* = 5,303; excluding those who deceased; interview duration: 84 minutes), 87.8% (*n* = 4,618; interview duration: 83 minutes), and 87.7% (n = 4,007; interview duration: 101 minutes), respectively. Unless indicated otherwise, rates of variables at baseline reflect lifetime occurrence and rates at T1 to T3 reflect interval (baseline-T1, T1–T2, and T2-T3) occurrence of approximately 3 years. Previous analyses established that any mental disorder in the 12 months preceding the first wave was not associated with overall attrition over the follow-up period (de Graaf, van Dorsselaer, Tuithof, & ten Have, 2013; De Graaf, van Dorsselaer, Tuithof, & Ten Have, 2018). The study was approved by the Medical Ethics Review Committee for Institutions on Mental Health Care and written informed consent was collected from participants at each wave.

Assessment of DSM-IV disorders

The following 13 CIDI, version 3.0, DSM-IV diagnoses were assessed: major depression, dysthymia, bipolar disorder, panic disorder, agoraphobia, social phobia, specific phobia, GAD, alcohol abuse and dependence, drug abuse and dependence and any clinical psychosis. For assessment of clinical psychosis, a psychosis add-on instrument based on the G section of previous CIDI versions was included. This add-on instrument consists of 20 psychotic symptoms corresponding to the symptoms assessed in a previous population survey in the Netherlands, NEMESIS, the precursor of NEMESIS-2 (Bijl, Ravelli, & van Zessen, 1998; de Graaf et al., 2010). Detailed descriptions of the specific psychotic experience items (PE) can be found in previous work using NEMESIS (Smeets et al., 2013) and NEMESIS-2 (van Nierop et al., 2012). At baseline, lifetime prevalence of PE was assessed. A clinician did a follow-up telephone interview when participants reported a psychotic symptom to assess whether this symptom was a true PE using questions from the Structured Clinical Interview for DSM-IV. Given similarities between CIDI self-reported and clinically validated PE, in terms of associations, predictive value and outcome (Bak et al., 2003; van der Steen et al., 2019; van Nierop et al., 2012), CIDI self-reported PE were used, thus increasing statistical power. PE were dichotomized (present vs. absent) consistent with previous work in NEMESIS and NEMESIS-2 (Pries et al., 2018; Radhakrishnan et al., 2019; van Rossum, Dominguez, Lieb, Wittchen, & van Os, 2011). Clinical psychosis was defined, consistent with previous work in this sample, as the combination of any psychotic symptom and use of antipsychotic medication or psychiatric hospitalization (Guloksuz et al., 2020).

Overfitting and multicollinearity

Given the use of up to 46 independent variables in the logistic regression model, we examined the possibility of overfitting and multicollinearity that may ensue. First, we computed the number of covariates remaining after elimination of multicollinear covariates, based on a maximum variance inflation factor (VIF) of 10, using the Stata subsetByVIF routine. This revealed that all 46 variables were retained, with a maximum remaining VIF of 6.30 and a mean VIF of 1.48. In order to address the possibility of overfitting, we used the Stata pmsampsize routine to compute the minimum sample size required for the development of a new multivariable prediction model using the criteria proposed by Riley and colleagues (Riley et al., 2019) . The sample size is computed to minimise overfitting and to ensure precise estimation of key parameters in the prediction model. For binary outcomes, there are three criteria: i) small overfitting defined by an expected shrinkage of predictor effects by 10% or less, ii) small absolute difference of 0.05 in the model's apparent and adjusted Nagelkerke's R-squared value, and iii) precise estimation (within +/- 0.05) of the average outcome risk in the population for a key time point of interest for prediction. This revealed that the required sample size was 4776 and therefore well within the NEMESIS-2 sampling frame.

Family history

Family history was assessed as a person-level binary variable in two stages, as described previously (Radhakrishnan et al., 2019). First, for participants who screened positive for the following CIDI psychiatric diagnoses, presence of the disorder in direct relatives was assessed at each interview wave: alcohol/drug abuse/dependence, depression/dysthymia, mania, and anxiety disorders (panic disorder, social phobia, agoraphobia, generalized anxiety disorder). More than 40% of the sample thus screened family history positive at any of the waves. Second, at T1, self-reported parental history of “severe anxiety or phobias”, “severe depression” and “delusions or hallucinations” were assessed in the entire sample: around 20% thus screened positive. Using these two sources of information, the proportion of the sample in which family history could be assessed (hereafter: ‘family history’) was 94%, as described previously (Radhakrishnan et al., 2019).

Table S1 – Distribution of 13 DSM-IV diagnoses, per interview wave, in NEMESIS-2 cohort

Wave*	N	Clinical psychosis	Bipolar disorder	Major depression	Dysthymia	Social phobia	Specific phobia	Panic disorder	Generalised anxiety disorder	Agora-phobia	Alcohol dependence	Alcohol abuse	Drug dependence	Drug abuse
		%												
1	6,646	1.0	1.2	20.0	1.4	9.3	8.3	3.9	4.6	1.7	1.7	12.0	1.6	1.0
2	5,303	0.5	0.7	6.9	0.4	2.0	3.1	1.5	1.6	0.5	0.9	2.6	0.4	0.5
3	4,618	0.3	0.7	6.6	0.3	2.2	2.9	1.5	1.7	0.5	0.8	2.5	0.4	0.3
4	4,007	0.4	0.9	7.4	0.5	2.1	3.2	1.5	2.1	0.5	0.7	2.0	0.3	0.4
Total	20,574	1.0	1.2	20.0	1.4	9.3	8.3	3.9	4.6	1.7	1.7	12.0	1.6	1.0

*Wave 1=life time prevalence; wave 2-4=interval prevalence

Table S2. Distribution of factors in social dimension of clinical characterization, per interview wave, in NEMESIS-2 cohort

Wave	N	Living alone	Single/ divorced	Unemployed	Low income	Low education	Status gap	Disability pension	Young age	Female sex	Ethnic minority	Urban residence	Children at home
		%											
1	6,646	21.0	58.0	7.6	32.0	32.0	17.0	5.2	24.0	55.0	8.8	43.0	43.0
2	5,303	20.0	62.0	7.9	26.0	30.0	24.0	5.0	17.0	55.0	7.3	44.0	44.0
3	4,618	20.0	65.0	8.6	22.0	30.0	21.0	4.9	12.0	55.0	6.8	43.0	43.0
4	4,007	21.0	66.0	7.5	18.0	29.0	16.0	4.9	7.6	56.0	6.5	50.0	41.0
Total	20,574	21.0	58.0	7.9	32.0	32.0	17.0	5.2	24.0	55.0	8.8	43.0	43.0

Table S3. Distribution of factors in clinical dimension of clinical characterization, per interview wave, in NEMESIS-2 cohort

Wave	N	Family history	Childhood adversity	Life event	Suicidality*	Slow at interview	High neuroticism	High extraversion
		%						
1	6,646	56.0	22.0	50.0	8.5	13.0	23.0	15.0
2	5,303	61.0	20.0	45.0	2.2	19.0	22.0	15.0
3	4,618	62.0	18.0	48.0	2.0	16.0	21.0	15.0
4	4,007	62.0	18.0	48.0	2.2	9.6	21.0	15.0
Total	20,574	60.0	20.0	48.0	4.2	15.0	22.0	15.0

*Wave 1=life time prevalence; wave 2-4=interval prevalence

Table S4. Distribution of factors in the somatic dimension of clinical characterization, per interview wave, in NEMESIS-2 cohort

Wave	N	Somatic disorder	High pain	High BMI	Sufficient movement	Smoking	Hearing impairment	Visual impairment
		%						
1	6,646	35.0	23.0	45.0	42.0	31.0	2.6	0.5
2	5,303	42.0	26.0	49.0	40.0	27.0	3.0	0.6
3	4,618	43.0	28.0	50.0	40.0	23.0	3.1	0.8
4	4,007	42.0	26.0	53.0	42.0	19.0	3.3	0.7
Total	20,574	40.0	26.0	49.0	41.0	26.0	2.9	0.6

Table S5. Distribution of symptom scores dimension of clinical characterization, per interview wave, in NEMESIS-2 cohort

Wave	Symptom dimension	Mean	SD	N
1	Psychosis	0.3	0.8	6646
	Anxiety	4.94	6.67	
	Depression	4.01	7.34	
	Mania	0.64	1.82	
2	Psychosis	0.14	0.56	5303
	Anxiety	1.92	4.2	
	Depression	1.52	4.93	
	Mania	0.37	1.32	
3	Psychosis	0.1	0.47	4618
	Anxiety	1.95	4.24	
	Depression	1.41	4.6	
	Mania	0.33	1.2	
4	Psychosis	0.1	0.46	4007
	Anxiety	1.91	4.26	
	Depression	1.52	4.72	
	Mania	0.27	1.03	

References

Alonso, J., Angermeyer, M. C., Bernert, S., Bruffaerts, R., Brugha, T. S., Bryson, H., . . . EsemeD/Mhedea Investigators - European Study of the Epidemiology of Mental Disorders Project. (2004). Sampling and methods of the European Study of the Epidemiology of Mental Disorders (ESEMeD) project. *Acta Psychiatrica Scandinavica Supplementum*(420), 8-20. doi:10.1111/j.1600-0047.2004.00326

Bak, M., Delespaul, P., Hanssen, M., de Graaf, R., Vollebergh, W., & van Os, J. (2003). How false are "false" positive psychotic symptoms? *Schizophrenia Research, 62*(1-2), 187-189.

Bijl, R. V., Ravelli, A., & van Zessen, G. (1998). Prevalence of psychiatric disorder in the general population: results of the Netherlands Mental Health Survey and Incidence Study (NEMESIS). *Social Psychiatry and Psychiatric Epidemiology, 33*(12), 587-595.

de Graaf, R., ten Have, M., Burger, H., & Buist-Bouwman, M. (2008). Mental disorders and service use in the Netherlands. Results from the European Study of the Epidemiology of Mental Disorders (ESEMeD) In T. Ustun & R. Kessler (Eds.), *The WHO World Mental Health Surveys: Global Perspectives on the Epidemiology of Mental Disorders* (pp. 388–405). New York: Cambridge University Press.

de Graaf, R., Ten Have, M., & van Dorsselaer, S. (2010). The Netherlands Mental Health Survey and Incidence Study-2 (NEMESIS-2): design and methods. *Int J Methods Psychiatr Res, 19*(3), 125-141. doi:10.1002/mpr.317

de Graaf, R., ten Have, M., van Gool, C., & van Dorsselaer, S. (2012). Prevalence of mental disorders and trends from 1996 to 2009. Results from the Netherlands Mental Health Survey and Incidence Study-2. *Social Psychiatry and Psychiatric Epidemiology, 47*(2), 203-213. doi:10.1007/s00127-010-0334-8

de Graaf, R., van Dorsselaer, S., Tuithof, M., & ten Have, M. (2013). Sociodemographic and psychiatric predictors of attrition in a prospective psychiatric epidemiological study among the general population. Result of the Netherlands Mental Health Survey and Incidence Study-2. *Comprehensive Psychiatry, 54*(8), 1131-1139. doi:10.1016/j.comppsych.2013.05.012

De Graaf, R., van Dorsselaer, S., Tuithof, M., & Ten Have, M. (2018). *Sociodemographic and psychiatric predictors of attrition in the third follow-up of the Netherlands Mental Health Survey and Incidence Study-2 (NEMESIS-2)*. Retrieved from Utrecht:

Guloksuz, S., Pries, L. K., Ten Have, M., de Graaf, R., van Dorsselaer, S., Klingenberg, B., . . . van Os, J. (2020). Association of preceding psychosis risk states and non-psychotic mental disorders with incidence of clinical psychosis in the general population: a prospective study in the NEMESIS-2 cohort. *World Psychiatry, 19*(2), 199-205. doi:10.1002/wps.20755

Pries, L. K., Guloksuz, S., Ten Have, M., de Graaf, R., van Dorsselaer, S., Gunther, N., . . . van Os, J. (2018). Evidence That Environmental and Familial Risks for Psychosis Additively Impact a Multidimensional Subthreshold Psychosis Syndrome. *Schizophrenia Bulletin, 44*(4), 710-719. doi:10.1093/schbul/sby051

Radhakrishnan, R., Guloksuz, S., Ten Have, M., de Graaf, R., van Dorsselaer, S., Gunther, N., . . . van Os, J. (2019). Interaction between environmental and familial affective risk impacts psychosis admixture in states of affective dysregulation. *Psychological Medicine, 49*(11), 1879-1889. doi:10.1017/S0033291718002635

Riley, R. D., Snell, K. I. E., Ensor, J., Burke, D. L., Harrell Jr, F. E., Moons, K. G. M., & Collins, G. S. (2019). Minimum sample size for developing a multivariable prediction model: PART II - binary and time-to-event outcomes. *Statistics in Medicine, 38*(7), 1276-1296. doi:https://doi.org/10.1002/sim.7992

Smeets, F., Lataster, T., van Winkel, R., de Graaf, R., Ten Have, M., & van Os, J. (2013). Testing the hypothesis that psychotic illness begins when subthreshold hallucinations combine with delusional ideation. *Acta Psychiatrica Scandinavica, 127*(1), 34-47. doi:10.1111/j.1600-0447.2012.01888.x

van der Steen, Y., Myin-Germeys, I., van Nierop, M., Ten Have, M., de Graaf, R., van Dorsselaer, S., . . . van Winkel, R. (2019). 'False-positive' self-reported psychotic experiences in the general population: an investigation of outcome, predictive factors and clinical relevance. *Epidemiol Psychiatr Sci, 28*(5), 532-543. doi:10.1017/S2045796018000197

van Nierop, M., van Os, J., Gunther, N., Myin-Germeys, I., de Graaf, R., ten Have, M., . . . van Winkel, R. (2012). Phenotypically continuous with clinical psychosis, discontinuous in need for care: evidence for an extended psychosis phenotype. *Schizophrenia Bulletin, 38*(2), 231-238. doi:10.1093/schbul/sbr129

van Rossum, I., Dominguez, M. D., Lieb, R., Wittchen, H. U., & van Os, J. (2011). Affective dysregulation and reality distortion: a 10-year prospective study of their association and clinical relevance. *Schizophrenia Bulletin, 37*(3), 561-571. doi:10.1093/schbul/sbp101
